# Preserved Excitatory-Inhibitory Balance of Cortical Synaptic Inputs following Deprived Eye Stimulation after a Saturating Period of Monocular Deprivation in Rats

**DOI:** 10.1371/journal.pone.0082044

**Published:** 2013-12-12

**Authors:** Giuliano Iurilli, Umberto Olcese, Paolo Medini

**Affiliations:** Department of Neuroscience and Brain Technologies, Istituto Italiano di Tecnologia, Genova, Italy; University of Southern California, United States of America

## Abstract

Monocular deprivation (MD) during development leads to a dramatic loss of responsiveness through the deprived eye in primary visual cortical neurons, and to degraded spatial vision (amblyopia) in all species tested so far, including rodents. Such loss of responsiveness is accompanied since the beginning by a decreased excitatory drive from the thalamo-cortical inputs. However, in the thalamorecipient layer 4, inhibitory interneurons are initially unaffected by MD and their synapses onto pyramidal cells potentiate. It remains controversial whether ocular dominance plasticity similarly or differentially affects the excitatory and inhibitory synaptic conductances driven by visual stimulation of the deprived eye and impinging onto visual cortical pyramids, after a saturating period of MD. To address this issue, we isolated visually-driven excitatory and inhibitory conductances by in vivo whole-cell recordings from layer 4 regular-spiking neurons in the primary visual cortex (V1) of juvenile rats. We found that a saturating period of MD comparably reduced visually–driven excitatory and inhibitory conductances driven by visual stimulation of the deprived eye. Also, the excitatory and inhibitory conductances underlying the synaptic responses driven by the ipsilateral, left open eye were similarly potentiated compared to controls. Multiunit recordings in layer 4 followed by spike sorting indicated that the suprathreshold loss of responsiveness and the MD-driven ocular preference shifts were similar for narrow spiking, putative inhibitory neurons and broad spiking, putative excitatory neurons. Thus, by the time the plastic response has reached a plateau, inhibitory circuits adjust to preserve the normal balance between excitation and inhibition in the cortical network of the main thalamorecipient layer.

## Introduction

Cortical circuits are extremely sensitive to modifications of the sensory environment, particularly during postnatal critical periods. MD is a classical paradigm of experience-dependent plasticity as it causes a dramatic loss of synaptic responsiveness through the deprived eye. Depression of visual responsiveness after MD can be accounted for by LTD-like mechanisms[1]. Available data suggest that the early loss of excitatory visual drive impacts more on excitatory neurons compared to inhibitory interneurons[2], [3] – but see[4]. Moreover, inhibitory drive from fast spiking interneurons to excitatory pyramidal neurons potentiates in layer 4 of the binocular V1 during the critical period[5]. Therefore, the excitatory-inhibitory balance of synaptic inputs to excitatory cells is initially shifted towards inhibition, thus favouring further loss of excitatory drive from the closed eye[2]. However, it remains unclear whether ocular dominance plasticity similarly or differentially affects the excitatory and inhibitory synaptic conductances driven by visual stimulation of the deprived eye and impinging onto visual cortical pyramids when the plastic is over, that is after a saturating period of MD[3], [6]. Recent work performed in mice suggests that monocular deprivation leads to a similar decrease of excitation and inhibition from the deprived eye. In contrast excitation from the non-deprived eye is preserved, while inhibition is decreased[7]. It is very important to check up to which degree this finding is preserved across phylogenesis. Rats display a stronger ocular dominance shift compared to mice ([8] vs [9]) and have a higher visual acuity[10], [11]. Importantly, the functional architecture of V1 in rats begins to display a tendency of visual cortical neurons to cluster vertically according to ocular dominance[12], [13], a first trace of ocular dominance patches that is absent in mice, even at the level of local microclustering of neurons[14]. Here we measured visually-driven inhibitory inputs to layer 4 pyramidal neurons and the spike output of the main class of inhibitory interneurons after a saturating period of MD in rat V1. Our results show that synaptic inhibition driven by stimulation of the closed eye decreases in parallel with the excitatory drive, in a way that preserves a normal excitatory-inhibitory balance.

## Materials and Methods

### Surgery and animal handling

All animal experiments have been done in agreement with the National Law and Ministry of Health. The Italian Institute of Technology Animal Care and Use Committee specifically approved this study (Authorization IIT IACUC 0221). Long Evans rats (aged P30-P32) were anesthetized with urethane (1.6 g/kg, i.p.). Dexamethazone (0.01 mg/kg, i.p.) was injected to prevent cortical and mucosal oedema. Oxygen was administered to the animal through a nose cannula, and body temperature was maintained at 37°C through a thermostatic blanket. Corneal and pinch reflexes, EEG, electrocardiogram and breathing rate were continuously monitored during the experiment. Animals were kept at deep (surgical level) anaesthesia and anaesthesia depth was maintained during experiments by means of additional doses of urethane (5–10% of the initial dose administered i.p.). Rats were mounted on a stereotaxic apparatus and a region of skull lying above V1 of about 4×4 mm was thinned until the underlying vasculature was clearly visible. An imaging chamber made with acrylic cement was built on the skull and filled with saline warmed at 37°C.

### Visual deprivations and eye protection

Rats were anesthetized with avertin (tribromoethanol solution; 1 ml/hg animal weight) and placed on a thermostatic blanket at P21. Eyelids were suture-closed with 6-0 surgical wire (Ethicon) under a surgical microscope with 3 mattress stitches. Control littermates were also anaesthetized. Ophthalmologic ointment containing hydrocortisone and gentamicin was applied during surgery. Animals showing any reopening during the deprivation period or with corneal opacities (revealed by ophtalmoscopic examination on the day of recording) were discarded. During experiments artificial tears were used to prevent corneal dehydratation and ophthalmological examination of the cornea, and the visible part of the lens through the pupils were performed to exclude opacities of the eye optics.

### Intrinsic Signal Imaging

A vasculature (“green”) image was acquired under 540 nm illumination before starting the imaging session. During imaging, the cortex was illuminated with monochromatic light with a wavelength of 630 nm. Images were acquired using a cooled 50 Hz CCD camera connected with a frame grabber (Imager 3001, Optical Imaging Inc, Germantown, NY, USA), defocused ca. 500–600 μm below the pial surface. Data frame duration was 200 msec and a spatial binning of 3×3 was applied over the images, which were 4.5×4.5 mm, with a pixel resolution of about 25 μm. Eye bulbs were kept fixed with metal rings adjusted so that the eye pupils projected at ca 55–57 degs with respect to the vertical meridian. Corneas were protected with artificial tears.

Squared spots of 20×20 degs were randomly projected in nine different positions and presented to the contralateral eye. A craniotomy was opened in correspondence to the spot position flanking the vertical meridian (between 5 and 25 deg of eccentricity) and at 20 deg of elevation for recordings in bV1. The spots displayed squared drifting gratings (duration: 8 sec, spatial frequency: 0.05 cycles/deg, speed: 2 cycles/sec, contrast: 90%, mean grating luminance: 19 cd/m^2^). Stimulus orientation was randomly alternated during the 8 sec of stimulation (every 45 deg, every sec), and stimuli were randomly interleaved with a full-field blank screen whose luminance was equal to the mean luminances of the grating (K0 blank). This same background surrounded the stimulus spot. Stimuli were displayed at 25 cm from the animal's eyes on a Sony G520 cathode ray tube 22″ monitor. Ten blank “first frames” were collected before stimulus onset.

All image frames obtained during stimulus presentation were divided by the average image of the first 10 frames acquired just before stimulus presentation[15]. The relative decrease of reflectance, averaged over the stimulus presentation period, was then outlined. The spot area was taken as the image area where the visually evoked decrease in reflectance was higher than 50% of the peak decrease. This region was then overlaid with the vasculature “green” image.

### *In vivo* whole-cell recordings

5–9 MΩ borosilicate patch pipettes filled with intracellular solution (in mM: 135 K gluconate, 10 HEPES, 10 Na phosphocreatine, 4 KCl, 4 ATP-Mg, 0.3 GTP, pH 7.2, 3 mg/ml biocytin, osmolarity 291 mOsm) were lowered perpendicularly to the pia applying ca 300 mmHg of positive pressure until about 500 µm below the surface of the cortex. At that point, positive pressure was lowered to 30 mmHg and cells were searched for in voltage clamp mode[16]. On approaching a cell, pressure was relieved and light suction was applied to allow gigaseal formation. After capacitance compensation, a ramp of negative pressure usually led to whole-cell configuration. Recordings were performed with an EPC10plus (HEKA, Germany) operated in bridge mode. The membrane potential (V_m_) signal was digitized at 20 kHz and acquired using the program Patchmaster (Heka Elektronic, Germany). Access resistance was repeatedly monitored, compensated for and ranged between 10–100 MΩ. Seal resistance was higher than 2 GΩ, and spike height and overall V_m_ were stable throughout recordings. No holding current was used. Recording durations ranged from 20 min to 2 hrs.

The resting V_m_ of neurons was measured as the modal value of the most negative peak in the V_m_ distribution during spontaneous activity (V_m_ during Down states). AP threshold was measured at the peak of the second derivative of the V_m_ trace[17]. Input resistance was measured by the steady voltage response to 300 msec duration hyperpolarizing current pulses (−100 pA). The AP width was measured as the width of the AP at half-maximal amplitude measured from threshold to peak.

The screen was positioned at 20 cm from the rat's eyes and centered on the spot position used during the intrinsic imaging session. Three degrees wide moving light bars (luminance: 20 cd/m^2^, background luminance: 3 cd/m^2^, angular speed: 45–55 deg/sec) of various orientations (every 45 deg) were separately presented to the two eyes by means of TTL-controlled, custom-made mechanical eye shutters. A period of 4 seconds between the end of one stimulus presentation and the beginning of the next one was set to prevent adaptation of responsiveness.

Data were analyzed using custom-made software written in IgorPro (Wavemetrics, USA) and Matlab ® (The MathWorks USA). For the analysis of subthreshold responses, APs were truncated using linear interpolation and sweeps were averaged over 20 presentations. Cells were first screened for their preferred orientation using light bars moving in various orientations (every 45 deg). To compare the response strength of the two eyes, only sub- and supra-threshold responses in the preferred orientation (and direction) were considered. PSP amplitude was computed with respect to the mean V_m_ during the interstimulus period. To compute the amplitude of visually evoked AP responses, mean spontaneous AP rates were subtracted to take into account differences of this parameter among cells. To compute peristimulus time histograms (PSTHs) of AP counts, 50 msec binning was applied. To quantify ocular preference, we computed an ocular dominance index (ODI) for every cells defined as (C−I)/(C+I), where C and I are the amplitudes of the peak PSP (or AP) responses for the contralateral and ipsilateral eye, respectively.

### Conductance estimates

The time course of changes in input resistance was measured by fitting the relation between the membrane potential, V_m_(t), and the injected current, I_inj_(t), at each instant t to equation
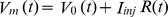
where R(t) is the input resistance at time t and V_0_(t) is a linear estimate of the membrane potential recorded without injected current[18]. I_inj_ is the injected current: 3 to 5 currents were injected (from −200 pA to 100 pA, mostly hyperpolarizing).

Synaptic conductances were estimated through a standard linear method based on the fundamental membrane equation[18], [19], [20].
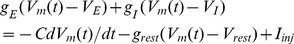
g_E_ and g_I_ are the excitatory and inhibitory synaptic conductance changes with respect to the resting, unstimulated state, respectively. V_E_ (0 mV) and V_I_ (−92 mV) are the reversal potentials for excitation and inhibition, respectively. The latter is intermediate between the equilibrium potentials of GABA_A_ and GABA_B_ channels according to the ionic composition of our intracellular solution. V_rest_ was the median V_m_ value during baseline (no visual stimulation) recording without current injection. V_m_ and dV_m_/dt are the recorded membrane potential and its derivative at any time point, respectively. C is the membrane capacitance that was calculated by
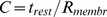
t_rest_ and R_memb_ are the time constant and the resistance of the cell membrane; they were calculated by least square fitting of the double exponential
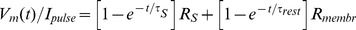
to the responses to 50 current pulses (I_pulse_ from −200 pA to 100 pA, 100 ms), each given before stimulus presentation. t_rest_ and t_s_ are the time constants of the membrane and of the patch electrode, respectively. R_s_ is the series resistance. To compensate for the series resistance, V_m_(t) was further corrected offline by:
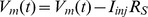



The accuracy of our least-square estimates was assessed by reconstructing the V_m_ waveform from the estimated g_E_ and g_I_ values. Our estimates accounted for 97.6±0.2% of the variance across our population of neurons.

1 mM QX314 was added to the pipette intracellular solution to block activation of voltage-dependent sodium (but also potassium[21], [22] and calcium[23]) voltage dependent conductances in an additional subgroup of cells (n = 9)

Finally, we calculated 95% confidence interval of gT, gE and gI by means of a bootstrapping strategy as explained in the following pseudoalgorithm:

REPEAT × 1000 {
 FOR each injected current level i
  Generate a new sample containing n Vm_i_ traces by sampling with repetition from the original sample of size n;
  Calculate the new average trace};
Estimate the new gT, gE and gI by solving the new system of equations;
Generate the distibutions of gT, gE and gI at each time point;

As a control for the quality of the clamp, we did voltage clamp experiments as in[24] in order to verify the presence of inward (excitatory) and outward (inhibitory) currents upon visual stimulation at the reversal potentials we used for our conductance estimates in current clamp. First, the preferred direction was found in current clamp conditions, as previously explained. During voltage clamp configuration, access resistance was automatically compensated, with an average compensation of 65%. The liquid junction potential was estimated to be +14 mV, and, although not compensated, was taken into account when clamping the cell at the reversal potential for excitation and GABAergic inhibition, which were set, respectively, at +14 mV and −80 mV.

### Extracellular Multiunit Recordings

Rats were implanted in the upper binocular visual cortex (see Intrinsic Signal Imaging section) with an array of four silicon-iridium tetrode probes for acute recordings (A4x1-Tet-3mm-150-121, Neuronexus Technologies, Ann Arbor, MI). Electrophysiological signals were acquired and digitalized at 32 kHz employing a Digital Lynx 4S recording system (Neuralynx, Bozeman, MT).

Signals were processed via custom off-line algorithms written in Matlab ® (The MathWorks Inc., Natick, MA). Signals were first average referenced to improve the signal-to-noise ratio[25] and then filtered in the 900 Hz − 9 kHz range for spike detection. Spikes were extracted by manually setting a threshold for each channel at approximately 3–4 times the standard deviation of background noise [26], [27], [28]. For each spiking event, 64 samples were extracted, from −0.65 ms to +1.29 ms around spike peak. Spike sorting was performed following the method detailed in [27]. For each recording channel, principal components analysis (PCA) was performed on spike waveform data, and the first 4 principal components (PCs) were retained for subsequent analyses, after checking that they accounted for at least 70% of all variance. Additional PCs were employed if such criterium was not met. Unsupervised clustering was then performed on the computed 16 PCs (usually 4 for each recording site), by employing split and merge expectation maximization (SMEM, [29]). In SMEM, a mixture of Gaussians is initially fitted to the dataset by following an expectation maximization procedure. Then, individual Gaussians are iteratively split and merged until convergence of the likelihood function to a maximal value is obtained. The SMEM algorithm has previously been successfully employed for spike sorting [27], [28]. The results of the clustering algorithm were always carefully checked, both visually and by means of quantitative indexes. Visual inspection was always necessary to discard clusters corresponding to artifacts and – eventually – repeat the whole procedure in case of poorly isolated units. Quality of isolation was verified for each cluster by computing a false positive/false negative index [27]. A set of simulated PC data points was generated using the parameters of the fitted mixture of Gaussians, thus knowing a priori to which cluster each point belonged. Then, each point was a posteriori assigned to a cluster, as if it was an experimental point, and the ratio of false positives and false negatives was computed. Clusters for which this ratio exceeded 0.05 were excluded as poorly isolated.

Several authors previously showed that it is possible to classify sorted cells into putative regular spiking or fast spiking neurons by analyzing the features of recorded action potentials [30], [31], [32]. Here we applied the methodologies developed by these authors in order to obtain a robust index of whether a cell is a regular or a fast spiking neuron. For each cell, we computed the average action potential and computed several pairs of feature: following [30] we measured the initial width – i.e. the duration of the initial wave – and the after-hyper-polarization width – i.e. the duration of the second wave of a spike; following [31] we computed the half-amplitude duration and the time between the negative and positive peak; finally, following [32], we measured two pairs of features, the ratio between the negative and positive peaks versus the end slope of the action potential, and the ratio between the negative and positive peaks versus the time between the two peaks. For each pair of features, we then classified the cells into two categories by performing a k-means clustering. Only those cells that were consistently classified as either NSUs or BSUs for all four pairs of features were assigned to the final categories; all remaining cells were left unclassified.

Spike trains were constructed for each direction and stimulated eye, by aligning spike times relative to stimulus onset for each trial, and computing 50 ms-binned PSTHs out of 40 trials per stimulus. The baseline firing rate was computed as the mean firing rate in the [−1500 0] ms window preceding stimuli, and the response of a cell to a given stimulus was computed as the difference between the maximal firing rate in the [0 2500] ms window after stimulus appearance and the baseline firing rate. Selection of preferred stimulus orientation and ODI calculation was performed as for intracellular recordings.

### Anatomy

At the end of the experiments, animals were transcardially perfused with 4% paraformaldehyde in 0.1 M phosphate buffer (pH 7.4). Brains were postfixed in the same solution overnight at 4°C. Then coronal sections of the brains were cut at 100 µm thickness using a cutting vibratome. Sections were then counterstained for cytochrome C[33], [34], byocitin-filled neurons were recovered with standard peroxidase histochemistry and slides were mounted with Mowiol and coverslipped. Cell identity was controlled at 10× and 20× magnification under a conventional brightfield microscope.

Verification of laminar depth for extracellular recordings was done by visualizing the track of the electrode, previously painted with DiI crystals dissolved in absolute ethanol, in Nissl-counterstained coronal sections.

### Statistical analysis

Normality was tested using both a Kolmogorov-Smirnov test and a Shapiro test. For normally distributed data, means ± s.e.m. are reported, otherwise medians are reported. Box plots represent the range (crosses), the 5–95 percentile (outer segments) and the 25–75 percentiles (box); the line inside the box is the median, and the square is the mean. The following comparisons have been done: normally-distributed data were compared with t-tests (when unpaired) or with paired t-tests when paired; non-normally distributed data were compared with Mann-Whitney Rank Sum test for unpaired comparisons.

## Results

### Loss of synaptic responsiveness to the deprived eye is larger than potentiation of open eye inputs

**Figure 1:**
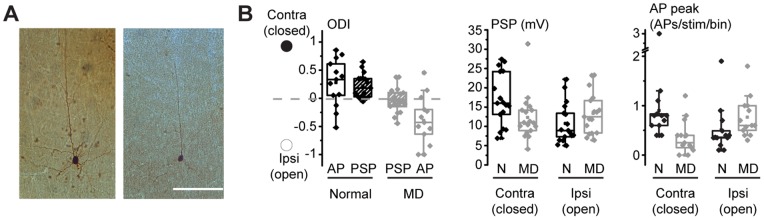
Sub- and supra-threshold effects of MD in 4RSNs of rat V1. **A**. Figure S1. Examples of anatomically recovered layer 4 star pyramids. 100 µm thick coronal sections were reacted for cytochrome C to reveal layer 4 and byocitin-filled cells were recovered with peroxidase-based histochemistry. Calibration bar: 100 µm. **B**. Left: the ocular preference shift –as assessed by the decrease of the ODI- caused by MD (P20–P30) was larger for AP responses (open boxes) compared to PSP responses (dashed boxes). Middle: at the level of PSP responses, depression of responsiveness to the deprived eye was larger (Mann-Whitney Rank Sum test, p<0.05) compared to potentiation of open eye responses (Mann-Whitney rank Sum test, p = 0.1). Right: at the level of AP responses, both depression of responses to the deprived eye and potentiation of open eye responses were significant (Mann-Whitney Rank Sum tests, p<0.05). For a more detailed analysis of date presented in this figure see also[41]

We isolated visually-driven excitatory and inhibitory conductances from regular spiking neurons in layer 4 (4RSNs – see Table 1), the layer where ocular dominance plasticity (ODP) is expressed shortly after the onset of MD in rodents[35], [36]. Neurons were at nominal depths – corrected for the penetration angle – corresponding to layer 4 (25^th^–75^th^ percentiles: 644 – 699 µm). Cells where biocytin-filled and when anatomical recovery was successful, we always found layer 4 star pyramids (Figure 1A), as in [35].

**Table 1:** Basic biophysical properties of 4RSNs.

	Control rats	MD rats (P21–P30)
Input Resistance (MΩ)	143.9±16.5	150.3±8.8
Resting Vm (mV)	−75.7±1.8	−77.3±2.0
Spontaneous APs (Hz)	2.44±0.44	1.48±0.39
AP half-width (ms)	1.07±0.05	1.04±0.09
AP threshold (mV)	−38.6±1.0	−37.6±1.9

± S.E.M. are given. No significant differences between Control and MD rats for all parameters (t-tests, p>0.2). Means

Rats were subjected to a saturating period of MD (P21–P30)[9], [37] and *in vivo* whole-cell recordings were targeted to the central-upper binocular visual field by intrinsic signal imaging. We compared sub- and supra-threshold responses to optimally oriented moving light bars independently presented to the two eyes[35]. The ocular preference of each neuron was quantified by computing its ocular dominance index (ODI), defined as (C−I)/(C+I), where C and I are the peak postsynaptic potential (PSP) or action potential (AP) responses driven by the contralateral and ipsilateral eyes, respectively. This index is +1 to -1 for neurons driven solely by the contralateral and ipsilateral eye, respectively, and is zero for perfectly binocular cells. In controls ODI values are biased in favour of the contralateral eye[9]. The ocular preference shift caused by MD was larger for AP responses (Figure 1B, left –open boxes; ODIs: 0.28±0.12 vs −0.39±0.12 for normal and MD rats, respectively; t-test, p<0.001) compared to PSPs (dashed boxes; 0.22±0.05 vs −0.03±0.05 for normal and MD rats, respectively; t-test, p<0.01). The ODI shift caused by MD was three-fold larger for AP responses compared to PSPs (0.25 vs 0.66). The separate analysis of the responses driven by the open and closed eye indicated that loss of PSP responsiveness to the contralateral, closed eye (Figure 1B, middle; medians: 16.0 vs 10.9 mV for normal and MD rats, respectively; Mann-Whitney Rank Sum test, p<0.05) was larger compared to input potentiation driven by the open eye (Figure 1B, middle, medians: 8.9 vs 12.5 mV for normal and MD rats, respectively; Mann-Whitney rank Sum test, p = 0.1). At the AP level (Figure 1B, right), both deprived eye response depression (medians: 0.8 vs 0.25 AP/stim/bin for normal and MD rats, respectively; Mann-Whitney Rank Sum test, p<0.01) and open eye response potentiation (medians: 0.36 vs 0.6 AP/stim/bin for normal and MD rats, respectively; Mann-Whitney Rank Sum test, p<0.05) were significant.

Thus, the ocular preference shift caused by MD is larger for AP responses compared to PSP responses, and loss of synaptic responsiveness from the closed eye is larger than potentiation of open eye synaptic inputs.

### Excitatory and inhibitory responses to amblyopic eye stimulation were similarly reduced

We wondered whether the balance between visually-driven excitation and inhibition is altered by 10 days of monocular deprivation. Although excitatory and inhibitory inputs largely overlap during visual responses, they could be set apart by measuring current responses at the reversal potentials for excitatory and inhibitory currents. However, pushing the membrane potential of a cell close to 0 mV strongly activates voltage-dependent conductances, that would distort a correct measurement of the inhibitory response. Hence, we adopted a similar strategy by holding the neuron at different membrane potentials but within a range that minimized that contamination. Importantly, we carefully checked the linear behaviour of the current-to-voltage response of the cells before calculating conductances. This was a critical step in order to employ the fundamental membrane equation to compute the visually-driven excitatory and inhibitory conductances, since this assumes a linear behaviour of the membrane (that is, a voltage-independence of synaptic conductances[18]). Crucially, as thoroughly discussed in[38], although such linear behavior is limited to a interval of membrane potential values spanning from −90 until −50 mV, current-clamp and voltage clamp recordings give rise to statistically undistinguishable excitatory and inhibitory conductance estimates *in vivo*.

**Figure 2:**
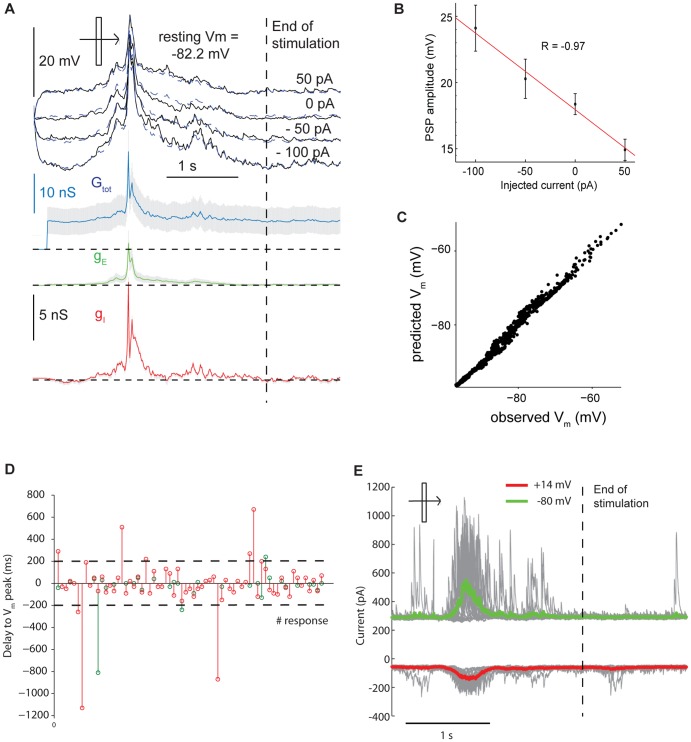
Estimate of visually-driven excitatory and inhibitory synaptic conductances. **A**. Visual responses to an optimally oriented moving light bar of a 4RSN recorded under 1 mM QX314 while injecting different steady currents. The black, continuous line are the recorded V_m_ values, whereas the blue, dashed trace shows the reconstructed V_m_ values obtained by inserting back the estimated g_E_ and g_I_ values into the fundamental membrane equation. The instantaneous total synaptic conductance is calculated based on the instantaneous slope of the current-voltage relation (G_tot_, blue). The time-dependent excitatory (g_E_, green) and inhibitory (g_I_, red) conductances are plotted below. Gray traces represent the 95% confidence intervals obtained by bootstrapping of the data (see Methods). Conductance measurements began after the response to the injected current was at steady state (after 200 ms). Resting conductances were calculated in absence of visual stimulation (dashed line: stimulus end). **B**. Visually-driven PSPs vary linearly with the injected current. Plot showing the linearity of the relationship between the amplitude of the visually-driven PSP response and the value of the injected current (r = −0.97) for a 4RSN (this plot refers to the example shown in Figure 2 of the Main Text). Means ± standard erros are shown. The median of the correlation coefficients for all the recorded neurons was −0.94 (25^th^–75^th^ percentiles: −0.88 − −0.99). **C**. Plot of the recorded *vs* reconstructed V_m_ values obtained by inserting back the estimated g_E_ and g_I_ into the membrane equation. The linearity of the cell and the accuracy of the V_m_ reconstruction is shown by the fact that data points align along the line of steepness 1 and intercept 0 in the plot. **D**. Temporal intervals between the peaks of g_E_ (green) and g_I_ (red) and that of the V_m_ response. For each cell, values for both contralateral and ipislateral responses are plotted. Note that in the vast majority of cases the conductance values have been obtained in close proximity of the V_m_ peak response (within 200 ms, dashed lines). **E**. Example of a voltage clamp recording (see Methods) following visual stimulation with a moving bar in the preferred direction. By clamping the cell at the reverse potential for inhibition (−80 mV when considering a liquid junction potential of approximately 14 mV) only excitatory currents can be seen (green: average response overlapped to single trials, shown in gray). Conversely, clamping the cell at the reversal potential for inhibition (+14 mV when considering the liquid junction potential) reveals the presence of inhibitory currents (red: average response overlapped to single trials, shown in gray).

**Figure 3:**
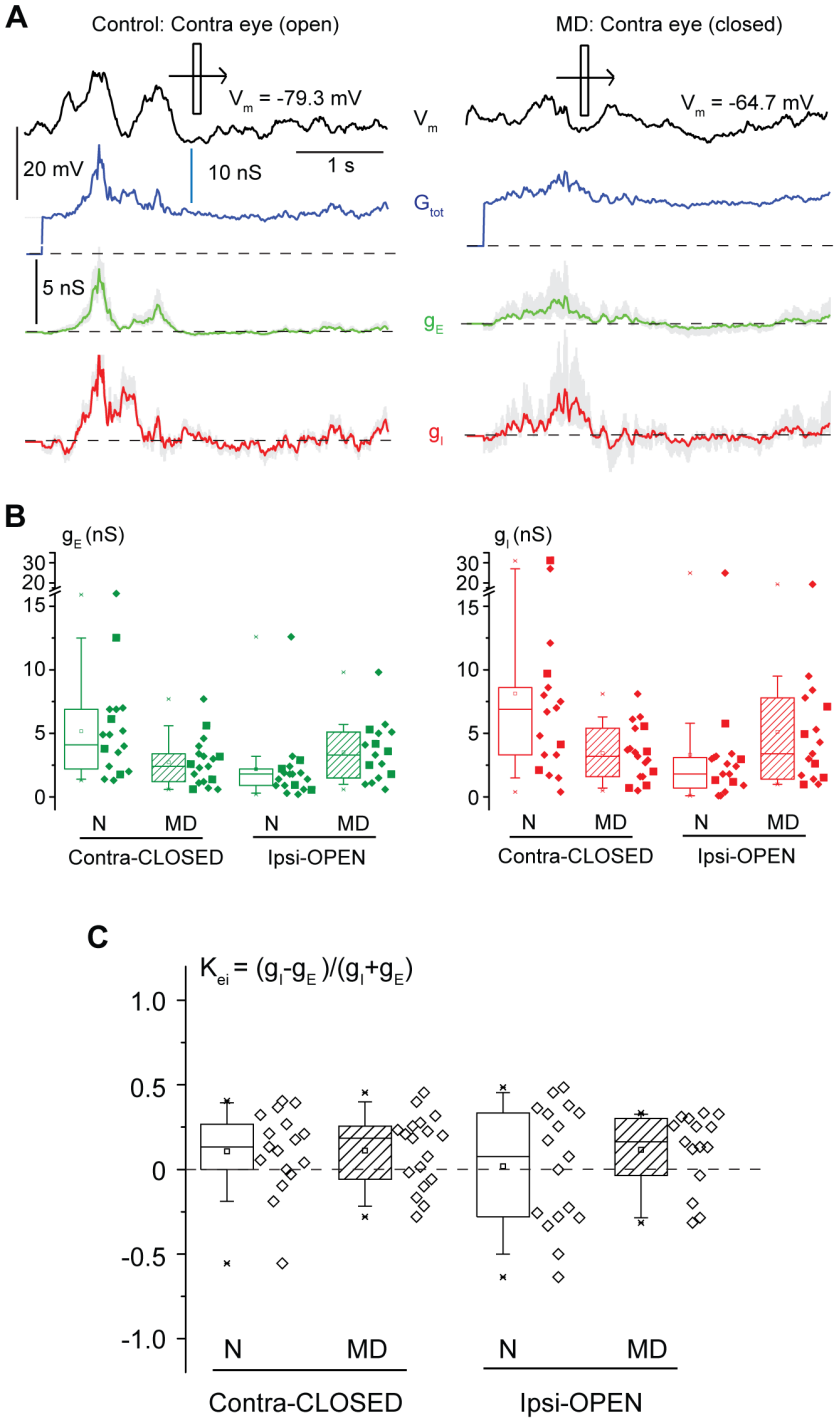
A saturating period of MD reduces both excitatory and inhibitory responses to closed eye stimulation. **A**. Examples of excitatory (g_E_, red) and inhibitory (g_I_, green) responses of 4RSNs (left: normal; right: MD) upon stimulation with optimally oriented light bars. The total membrane conductance (G_tot_) is shown in blue and the V_m_ response in absence of current injection is in black (top traces). Dotted lines: 0 nS. Gray shadow: 95% confidence intervals of the g_E_ and g_I_ estimates obtained by bootstrap analysis. **B**. Amplitudes of the visually-driven g_E_ (green) and g_I_ (red) responses in normal (open boxes) and MD (dashed boxes) rats. MD reduced both excitatory and inhibitory conductances upon contralateral eye (closed in MD rats) stimulation and increased both excitatory and inhibitory conductances upon ipsilateral eye (open in MD rats) stimulation (Mann-Whitney Rank Sum tests, p<0.05). **C**. The excitatory-inhibitory balance of visually-driven responses, expressed by the K_EI_ index, was not affected by MD (dashed boxes vs open boxes) for both contralateral and ipsilateral responses (Mann-Whitney Rank Sum tests, p>0.7).

To estimate synaptic conductances we measured the membrane potential (V_m_) response to visual stimulation while injecting steady, mostly hyperpolarizing currents (to minimize activation of voltage-dependent conductances -see example of Figure 2; n = 35 for contralateral eye conductances, n = 32 for ipsilateral eye conductances; 14 rats in the control group and 15 in the MD group), as in [19], [20]. In addition, 1 mM QX314 was added to the pipette intracellular solution in a subgroup of cells (n = 9) to block activation of sodium voltage-dependent conductances (but also potassium[21], [22] and calcium[23] conductances). Conductance estimates were statistically indistinguishable for cells recorded with and without QX314 (Mann-Whitney Rank Sum tests, p>0.2; QX314-data are the squares in Figure 3B), in agreement with previous works showing that the derivation of conductance values is not different when QX314 was either present or absent[20]. Moreover, the r^2^ values computed for the regressions between injected currents and PSP amplitudes were not significantly different for internal solutions with and without QX314 (medians 0.96 vs. 0.88 respectively, Wilcoxon rank sum test, p = 0.1). The membrane conductance (G_tot_, blue in Figures 2 and 3) was calculated by measuring the inverse of the slope of the voltage-to-current relation at each time point. The decomposition of the synaptic conductance into its excitatory (g_E_) and inhibitory (g_I_) components was done using the fundamental membrane equation. Due to conservation of the current, the injected current (I_inj_) is equal to the capacitative current (I_C_) and the resistive current across the membrane (I_R_):
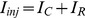
(1)where 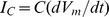
and 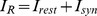 being C the membrane capacitance – calculated by applying small hyperpolarizing pulses – and *d*V_m_/*d*t the derivative of the membrane potential (V_m_) over time. I_rest_ and I_syn_ are the currents flowing through the resting and synaptic conductances, respectively. I_syn_ is the sum of excitatory and inhibitory currents. As each current can be espressed as the product of the respective membrane conductance by its driving force, equation (1) becomes:
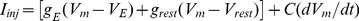
(2)where V_E_ and V_I_ are the known equilibrium potentials for excitatory and inhibitory currents, V_rest_ is the mean resting V_m_ measured in absence of visual stimulation, and g_E_, g_I_ and g_rest_ are the excitatory, inhibitory and resting conductances, respectively. g_rest_ was taken as the mean value of the instantaneous conductance measured in absence of visual stimulation. Thus, the visually-driven changes in excitatory and inhibitory conductances (green and red traces, respectively, in Figures 2 and 3), can be obtained by injecting at least two current levels and solving the resulting linear equation system by least-square fitting (see Methods). Experimental results did not change when the equilibrium potentials V_E_ and V_I_ were offset by ±10 mV (see Table 2). Importantly, this was true for the equilibrium potential V_I_. In the case of inhibition, we did not attempt to disambiguate between GABAA and GABAB receptor-mediated currents (as in e.g. [18], [19], [20], [39]). Of course, the relative contribution of GABAA- and GABAB-mediated currents might have been different by offsetting V_I_ by ±10 mV, but the fact that we came to similar conclusions indicated that such a dissection does not affect the quantification of the excitatory-inhibitory balance during visual responses. For our sample of neurons the amplitude of the PSP response varied linearly with current values, indicating the goodness of the linearity assumption (Figure 2B). Also, to further estimate the accuracy of our least-square solutions, we re-derived the V_m_ for each time point by inserting back in equation (2) the g_E_ and g_I_ values. Nonlinearities would be evident as a mismatch between actual and predicted V_m_ values (continuous and dotted lines in Figures 2A and 3A). The quality of the V_m_ trajectory reconstruction is also shown by the fact that in a plot displaying the actual and predicted V_m_ values data points distribute along the line with slope equal to 1 (Figure 2C). The amount of variance accounted for by the model in the example was 98.2%, and averaged 97.6±0.2% across neurons.

**Table 2:** Effects of offsetting equilibrium potentials V_E_ and V_I_ on g_E_ and g_I_ estimates.

	V_E_	V_I_	V_E−_	V_I−_	V_E+_	V_I+_
Contra N	4.5 (2.2–6.9)	6.9 (3.3–9.7)	5.9 (2.7–8.6)	5.5 (2.5–8.2)	3.4 (1.8–5.2)	8.3 (4.1–11.2)
Contra MD	2.4 (1.2–3.4)	3.3 (1.6–5.4)	3.1 (1.7–4.2)	2.7 (1.3–4.5)	1.6 (0.8–2.7)	4 (2–6.4)
Ipsi N	1.8 (0.9–2.4)	1.8 (0.5–3.1)	2.0 (1.2–3.1)	1.3 (0.4–2.6)	1.4 (0.6–1.9)	2.2 (0.9–3.5)
Ipsi MD	3.3 (1.6–5.1)	3.4 (1.5–7.6)	3.9 (1.9–6.6)	2.8 (1.2–6.3)	2.7 (1.2–3.5)	4 (1.8–8.9)

^th^–75^th^ percentiles). All comparisons along any column between normal (N) and MD rats were significant (Mann-Whitney Rank Sum tests, p<0.05). V_E_ and V_I_ are the estimated equilibrium potentials for excitation and inhibition (see methods section), V_E+_ and V_I+_ are V_E_ and V_I_ offset by +10 mV; V_E−_ and V_I−_ are V_E_ and V_I_ offset by −10 mV. Data are reported as follows: median (25

Finally, to further verify that our method was correctly showing the presence of both excitatory and inhibitory currents upon visual stimulation, we performed some voltage clamp experiments (as in [24]; n = 4 from 4 mice; see Methods and Figure 2E). By clamping cells at the estimated reversal potentials for either excitation or inhibition, we were able to demonstrate that visual stimulation elicited, respectively, outward and inward currents, respectively.

**Figure 4:**
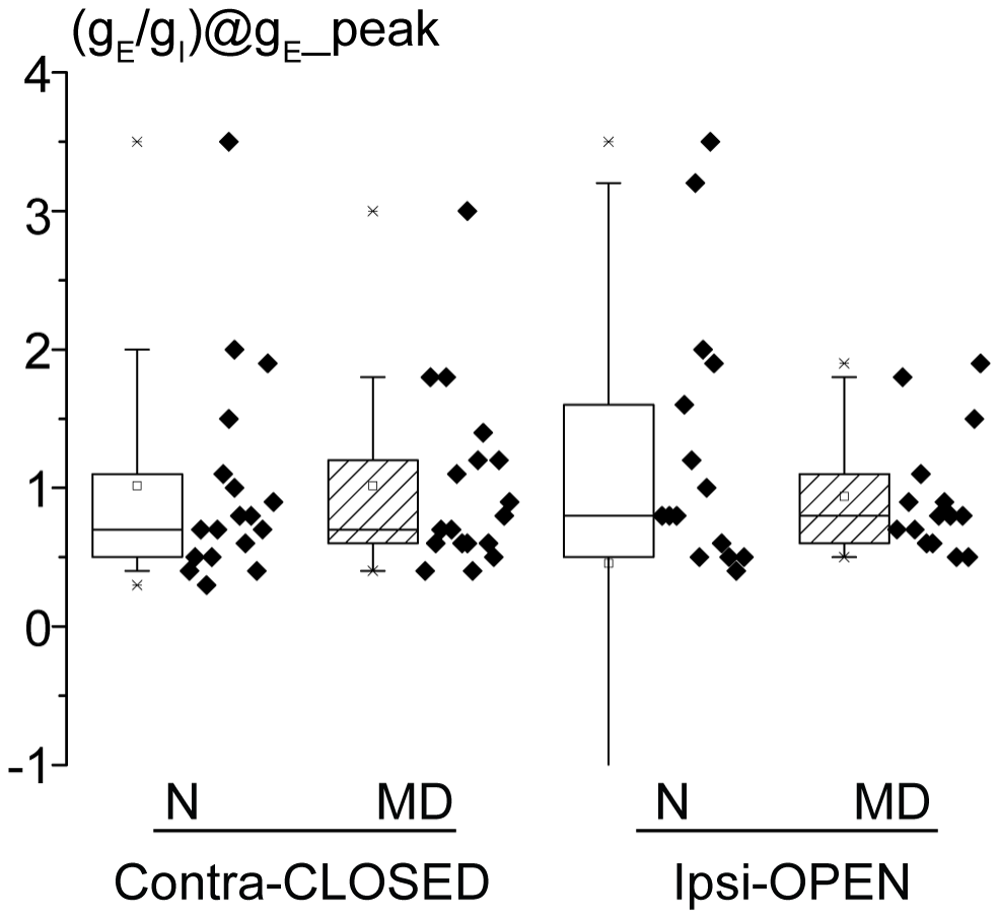
MD does not alter the ratio of excitation and inhibition measured at the excitation peak. The values of the ratio between g_E_ and g_I_ measured at the peak of the excitatory conductance are plotted for each experimental group. MD does not significantly modify the ratio values for both contralateral (left) and ipsilateral (right) eye responses (Mann-Whitney Rank Sum tests, p>0.6).

To relate the changes in visually-driven excitation and inhibition caused by a saturating period of MD to those of subthreshold and suprathreshold ocular dominance measured above, we measured the peak amplitudes of g_E_ and g_I_. Conductances peaked in proximity to the V_m_ visual responses (Figure 2D; delay from V_m_ peak to g_E_ peak, median: 15 ms, delay from V_m_ peak to g_I_ peak, median: 25 ms). The examples of Figure 3A show that after a saturating period of MD, for visual responses measured through the contralateral (deprived) eye, both the g_E_ and g_I_ peak values were decreased. This is quantified in Figure 3B, which shows a significant drop in visually-driven g_E_ and g_I_ values at population level (g_E_: medians 4.1 vs. 2.4 nS for control and MD rats; Mann-Whitney Rank Sum test, p<0.05; g_I_: medians 6.9 vs. 3.3 nS for control and MD rats; Mann-Whitney Rank Sum test, p<0.05). We expressed the excitatory-inhibitory balance of visual responses as the contrast between excitation and inhibition, defined as K_EI_ = (g_I_ − g_E_)/(g_I_+g_E_), an index that varies from +1 to −1 for visual responses dominated only by inhibition and excitation, respectively. For the closed eye in MD rats, the similarity in the drop of excitatory and inhibitory conductances caused by MD was reflected in the fact that K_EI_ values were similar for the visual responses driven by the contralateral eye in control and MD rats (Figure 3C, left; 0.13±0.06 vs 0.11±0.05 for normal and MD rats, t-test, p = 0.9). The same held for the ratio between g_E_ and g_I_ at the peak of g_E_ [40]; Figure 4, left; medians: 0.75 vs 0.75 for control and MD rats, Mann-Whitney Rank Sum test, p = 0.8).

Excitatory conductances driven by ipsilateral eye stimulation (open in MD rats) increased upon a saturating period of MD (Figure 3B, left; medians: 1.8 vs. 3.3 nS for control and MD rats; Mann-Whitney Rank Sum test, p<0.05). Ipsilaterally-driven inhibitory conductances were also increased (Figure 3B, right; medians: 1.8 vs. 3.4 nS for control and MD rats; Mann-Whitney Rank Sum test, p<0.05). Thus, the excitatory/inhibitory balance of the open eye responses did not change upon a saturating period of MD (median K_EI_: 0.05 vs. 0.14 for control and MD rats; Mann-Whitney Rank Sum test, p = 0.6, see Figure 3C, right; medians of the ratio between g_E_ and g_I_ at the peak of the g_E_: 0.8 vs. 0.8 nS for control and MD rats; Mann-Whitney Rank Sum test, p = 0.7 –see Figure 4, right).

**Figure 5:**
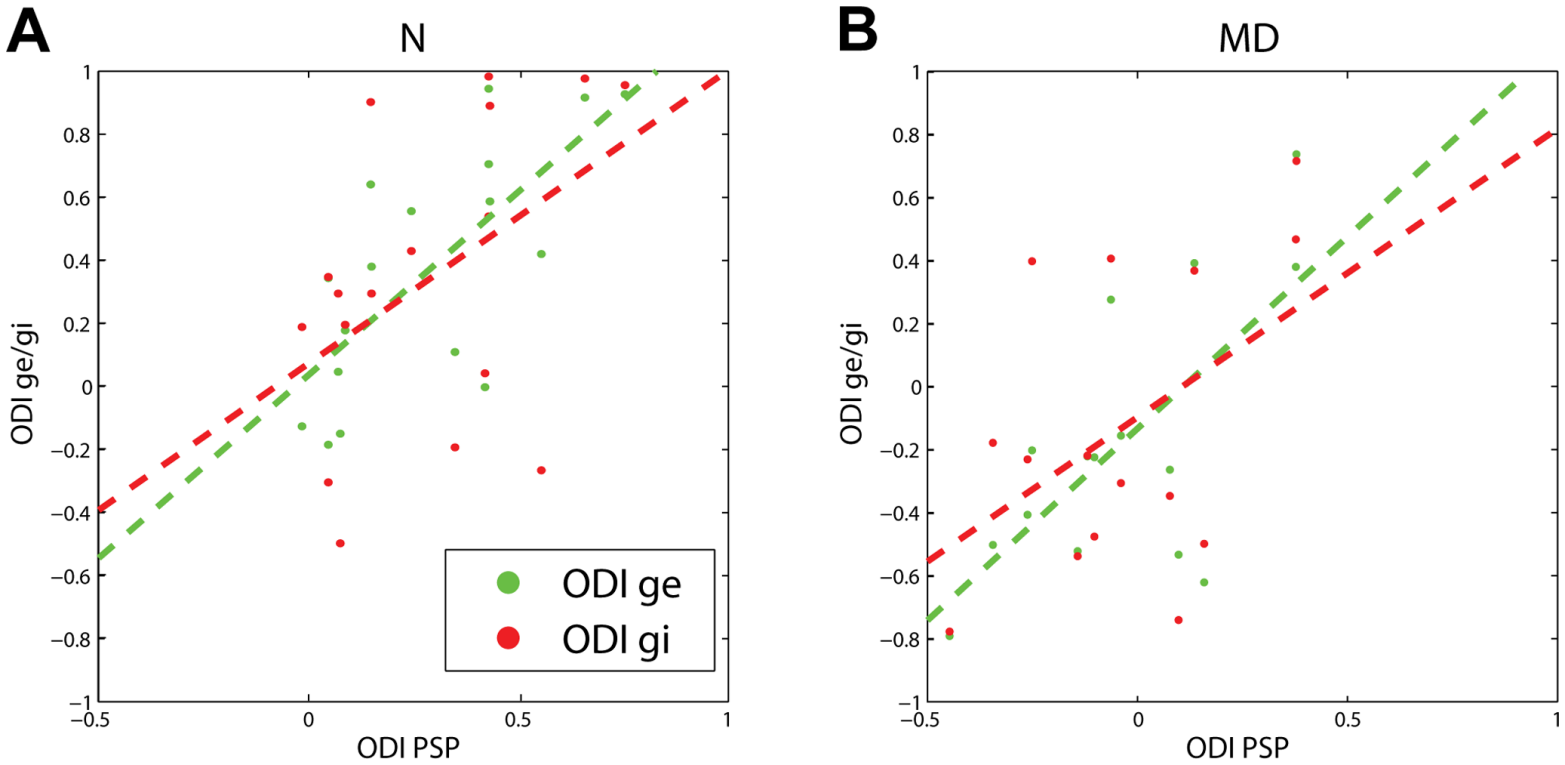
Relationship between synaptic and conductance-based ODIs. **A**. Raster plot showing the relationship between synaptic ODIs (ODI PSP) and ODIs for excitatory and inhibitory conductances (respectively ODI ge, in green and ODI gi, in red), for animal in the control (N) group. Dashed lines indicate the result of the linear fit between synaptic and conductance-based ODIs (ODI-ge vs. ODI-PSP: r = 0.73, p<0.01; ODI-gi vs. ODI-PSP: r = 0.47, p = 0.06). **B**. Same as in A for rats in the MD group (ODI-ge vs. ODI-PSP: r = 0.67, p<0.01; ODI-gi vs. ODI-PSP: r = 0.47, p = 0.06).

We next attempted to quantify the relation between the ocular preference of synaptic, subthreshold responses and that of excitation and inhibition. As the relationship between PSPs and APs following MD has been explored in previous studies[35], [41], we focused on the link between g_E_, g_I_ and PSPs. In particular we investigated whether ocular preference at the PSP level was more influenced by either the relative strength of the excitation driven by the two eyes or by the relative strength of inhibition, and we did this separately for control and MD rats. In both groups of animals, the ODI of synaptic (PSP) responses was significantly related to the ODI of excitatory responses (Figure 5, green traces; for control animals: r-value = 0.73, p-value<0.01; for MD animals: r-value = 0.67, p-value<0.01). However, the same relationship was weaker and barely significant for inhibitory conductances (Figure 5, green traces; for control animals: r-value = 0.47, p-value = 0.06; for MD animals: r-value = 0.47, p-value = 0.06). Thus, on a single cell basis, the main synaptic determinant of their ocular preference is the relative strength of the excitatory inputs coming from the two eyes rather than that of the inhibitory inputs.

Thus, when the effect of MD is complete, the excitatory-inhibitory balance of visually-driven responses is similar to that observed in controls. This holds for both contralateral, deprived-eye depressed response and for ipsilateral, open-eye response.

### Similar ocular dominance plasticity of extracellularly isolated excitatory and inhibitory neurons

**Figure 6:**
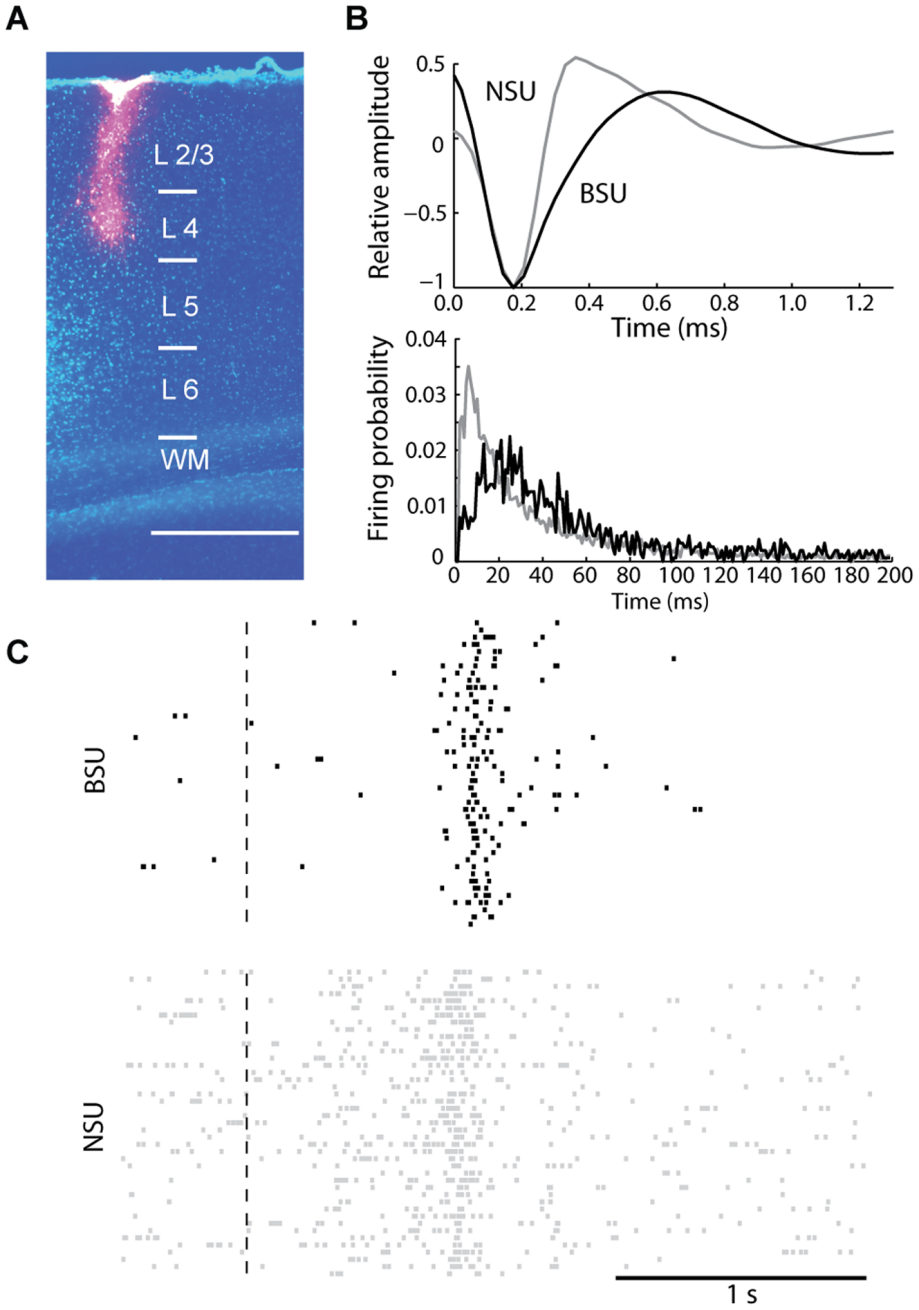
A BSU and a NSU isolated with spike sorting from multiunit recordings in layer 4. **A**. Nissl counterstained coronal section in a rat recorded with an electrode coated with DiI (red) consisting of 4 horizontally aligned tetrodes located at the tip (point of maximal penetration). Bar. 600 µm. **B**. Example of an extracellularly isolated BSU (black) and NSU (gray). Top plot: mean AP waveforms; bottom plot: interspike interval distributions. Both plots were obtained taking into account only APs occurring in absence of visual stimulation. Note the narrower AP shape of the NSU and that the interspike interval distribution of the NSU is skewed towards shorter values. **C**. Examples of raster plots recorded from a BSU (top, black) and a NSU (bottom, gray) when visual stimulation was presented. Dashed line indicates the motion onset of the light bar on the screen. Note the higher spontaneous activity for the NSU compared to the BSU.

**Figure 7:**
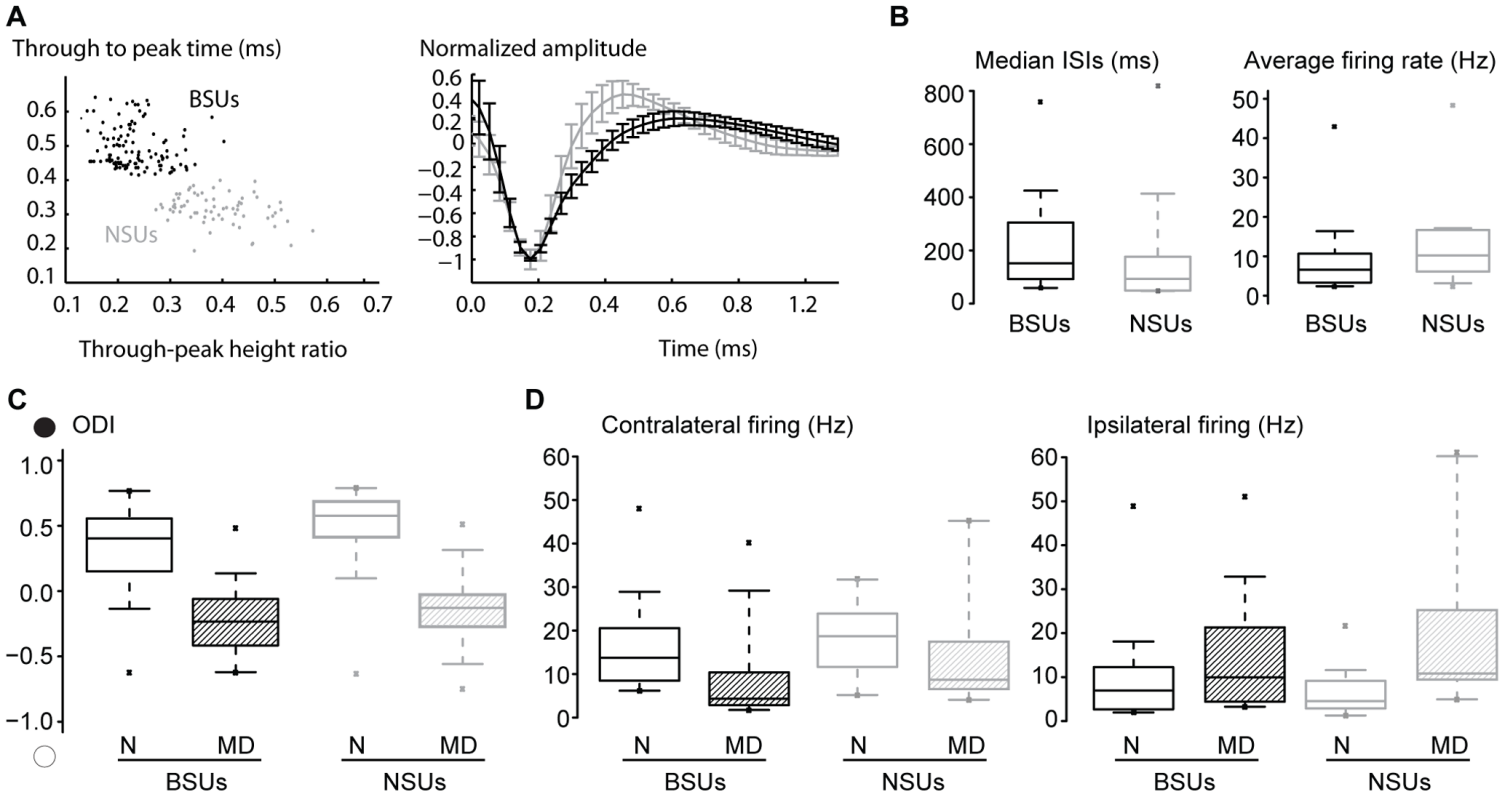
MD caused a comparable ocular dominance plasticity in extracellularly isolated, putative excitatory and inhibitory units. A. Outcome of the sorting of BSUs and NSUs from multiunit extracellular recordings in terms of AP waveform parameters. Right: average±s.e.m. AP shape of isolated BSUs (black) and NSUs (gray). Note the shorter AP duration and deeper afterhyperpolarization for NSUs. Left: plot showing the through-to-peak time vs through-peak height ratio of single isolated units. Note the clusters of the BSUs (black dots) and NSUs (gray dots). B. Spontaneous firing rates of NSUs were higher compared to BSUs. Median ISIs are plotted on the left, averaged firing rates on the right (Mann-Whitney Rank Sum tests, p<0.01). C. MD caused a significant drop of ODIs for both BSUs (black) and NSUs (gray) [t-tests, p<0.001]. D. Both depression of responses to the contralateral eye (left, closed in MD rats) responses and potentiation of responses to the ipsilateral eye (right, open in MD rats) were significant for both BSUs (black) and NSUs (gray) – Mann-Whitney Rank Sum tests, p<0.05.

Given the perisomatic distribution of the GABAergic synapses provided by parvalbumin-positive interneurons, the inhibitory conductances we measured from our somatic recordings are presumably largely contributed by these cells, which represent the majority of cortical interneurons in rat V1[42]. Two-photon-targeted recordings from interneurons in layer 4 of rats are prevented by depth limitations and by lack of rat transgenic lines with labeled parvalbumin cells. Thus, we exploited the fact that parvabumin-positive cells have a fast-spiking electrophysiological phenotype and hence can be identified in multiunit recordings based on their characteristic AP waveform and frequency as narrow-spiking units (NSUs, [30], [31], [32]). Conversely, putative excitatory cells are characterized by lower firing rates and a broader spike shape (broad spiking units, BSUs) - a classification that is 90% accurate[43]. Thus, we investigated whether putative inhibitory, NSUs are differentially sensitive to a saturating period of MD compared to putative excitatory, BSUs in layer 4 of rat V1. For this aim, we inserted extracellular tetrodes in layer 4 and then isolated single units by spike sorting (Figure 6). For each isolated unit, we computed the average AP waveform and computed several pairs of features: following [30] we measured the initial AP duration and the after-hyperpolarization duration; following [31] we computed the half-amplitude duration and the time between the negative and positive peaks; following [32] we measured two pairs of features, the ratio between the negative and positive peaks versus the AP end-slope, and the ratio between the negative and positive peaks versus the interpeak-interval. For each pair of features, we then classified the cells into two categories by performing a k-means clustering (see Method sections). In order to be classified as NSU or BSU a cell had to lie into the same cluster for all four pairs of features. Figure 7A (left) plots the AP-to-afterhyperpolarization interval *vs*. the AP peak-to-afterhyperpolarization amplitude ratio for all NSUs (n = 58) and BSUs (n = 96). Averaged AP shapes of NSUs and BSUs are shown in Figure 7A, right: NSUs have shorter AP durations and deeper afterhyperpolarization compared to BSUs, in agreement with the above-cited works. Firing patterns of NSUs and BSUs (measured in absence of visual stimulation) were also different. Median interspike interval (ISI) values were significantly shorter for NSUs compared to BSUs (Figure 7B, left; medians: 106.1 vs. 163.2 ms, respectively; Mann-Whitney Rank Sum test; p<0.01). As expected, average spontaneous firing rates of NSUs were higher compared to BSUs (Figure 7B, right; medians: 10.5 vs. 6.6 Hz, respectively; Mann-Whitney Rank Sum test, p<0.01). Conversely, only a trend was found for visual responses (expressed as the difference between peak and baseline firing rates, see Materials and Methods), with NSUs responding slightly more than BSUs (medians: 18.7 vs. 13.7 Hz for NSUs and BSUs respectively, Wilcoxon Rank Sum test, p = 0.09). Of relevance, whether fast-spiking NSUs and regular-spiking BSUs respond differently to the same stimulus remains controversial in the literature (for example, similar responsiveness has been found in[44], [45]). In conclusion, our clustering algorithm isolated two different neuronal populations, such as BSUs and NSUs, based on AP waveform features, that were statistically distinguishable also based on different, unrelated parameters (spontaneous firing pattern properties).

A saturating period of MD shifted the ocular preference of both BSUs (n = 47 in 7 controls and n = 49 in 5 MD rats) and NSUs (n = 37 in controls and n = 21 in MD rats; Figure 7C; mean ODIs for BSUs: 0.34±0.05 vs. −0.24±0.04, for NSUs: 0.49±0.05 vs. −0.12±0.07 for normal and MD rats, respectively; t-tests, p<0.001). This shift was similar for both cell types (difference of the mean ODI for BSUs and NSUs are 0.58 and 0.62, respectively). We next separately analyzed the responses to contralateral (deprived) and ipsilateral (open) eye stimulation in BSUs and NSUs. Contralateral (closed eye) responses were significantly depressed for both BSUs (Figure 7D, left -black; median peak responses with respect to baseline firing rates: 13.7 vs. 4.3 Hz for normal and MD rats, respectively; Mann-Whitney Rank Sum Test, p<0.001) and NSUs (Figure 7D, left -gray; median peak responses with respect to baseline firing rates: 18.7 vs. 8.7 Hz for normal and MD rats, respectively; Mann-Whtney Rank Sum test, p<0.05). Ipsilateral (open) eye input potentiation occurred both in BSUs (Figure 7D right -black, medians: 7.0 vs. 9.9 Hz for normal and MD rats, respectively; Mann-Whitney Rank Sum Test, p<0.05) and in NSUs (Figure 7D, right -gray, medians: 4.6 vs. 10.8 for normal and MD rats, respectively; Mann-Whitney Rank Sum test, p<0.001).

Thus, a saturating period of MD similarly shifts the ocular preference of both putative excitatory and inhibitory neurons. The neuronal populations undergo both depression of responses to the deprived eye and potentiation of responses to the open eye.

## Discussion

Here we showed that a saturating period of visual deprivation does not differentially affect excitatory and inhibitory conductances impinging on pyramidal neurons in layer 4. Indeed, we found that - when the plastic response in V1 has reached a plateau after one week of MD[35], [37] – visually-driven synaptic excitation and inhibition from the deprived eye are similarly reduced. Thus, the *balance* between visually-driven excitatory and inhibitory conductances onto layer 4 excitatory cells remained similar to controls. This is consistent with the view that MD may cause only a *temporary* imbalance between excitation and inhibition, restricted to the first two days after eye closure. Indeed, interneurons do not shift their ocular preference after only two days of MD in binocular V1 [2], [3] – but see[4], and synapses between fast-spiking units and pyramidal cells in layer 4 potentiate upon a brief MD[5]. Conversely, following a saturating period of MD, we found that fast-spiking inhibitory neurons undergo similar ocular dominance plasticity compared to putative excitatory units, as in[2], [3]. The temporary imbalance between excitation and inhibition immediately after MD could promote plasticity in excitatory cells by accelerating their loss of responsiveness to the deprived eye, the main response to MD in rats[35]. Indeed, temporarily maintaining normal levels of inhibition could further decrease synaptic efficacy from the deprived eye onto excitatory neurons, by Hebbian mechanisms. Of relevance, results similar to those presented here were recently reported in mice[7], employing a voltage-clamp paradigm. MD in mice indeed leads to a similar reduction in excitatory and inhibitory conductances from the deprived eye. Conversely, while excitatory conductances from the non-deprived eye do not show significant changes, inhibitory conductances are reduced. This is different from what we reported in rats, where excitation and inhibition change in parallel for both the deprived and the non-deprived eye. Since the two techniques for extracting in vivo synaptic conductances have been demonstrated to yield undistinguishable results (see Figure 10B of[38]), such discrepancy must therefore be due to species-specific differences. The discrepancy concerns very probably more the fact that the two species seems to have a different degree of plasticity when potentiation of the spared, open eye responses is concerned. Indeed, when we compare layer 4 recordings in mouse and rat V1, it is evident that the quantitative degree of potentiation is more robust in mice[46] as compared to rats[35], [41]. Also, during adulthood, MD induces a detectable shift of ocular preference by potentiating open eye inputs in mice[47] -possibly through a reduced inhibitory drive as documented by Ma et al [48], whereas a similar open eye input potentiation has not been documented to occur in the adult rat V1[37].

The potential role of inhibitory input plasticity following a saturating period of MD remained unclear. Previously, the role of inhibition in ODP observed after MD has been addressed pharmacologically, by extracellular micro-iontophoresis or intracellular perfusion with GABA blockers, with not fully consistent results. Bicuculline microiontophoresis in cat V1 produced variable results with regard to the percentage of cells that showed unmasking of deprived eye responses[49], [50], [51]. Moreover, iontophoretic GABA blockade broadens receptive fields and renders V1 neurons more binocular already in controls[49], [52]. Finally, a recent study in rodents failed to find consistent effects of intracellular GABA blockade in a saturating period of MD onto AP responses at population level[3]. Thus, although only GABA blockade experiments might be able to directly address the role of inhibition in the loss of responsiveness to closed eye stimulation, such experiments are affected by serious limitations ang gave rise to some degree of controversy. Here we provided *in vivo* measurements of changes in visually-driven excitatory and inhibitory conductances following a saturating period of MD. We found that the strength of *both* excitatory and inhibitory inputs to layer 4 excitatory cells decreased following a saturating period of MD, resulting in an ultimate re-balancing of excitation and inhibition. Thus, experience-dependent plasticity of excitatory and inhibitory systems appears comparable.

It is also important to stress that it is difficult to draw conclusions on whether such a rebalancing of the inhibition-excitation ratio upon a full week of MD plays a role in the loss of visual acuity (amblyopia) observed after more prolonged periods of MD even in rodents[10], [11], [37]. Indeed, albeit a full week of MD causes a maximal shift of ocular preference when compared with much longer deprivation times (several weeks - compare[9] with[37]), there are currently no indications on whether the duration of MD we used is long enough to produce some degree of amblyopia. In addition, the neural mechanisms underlying the ocular dominance shift and those underlying amblyopia might also be different[53], [54].

Because *in vivo* whole-cell recordings are mostly obtained from cell somatas[16], our estimates of inhibitory conductances are presumably biased in favour of perisomatic inhibitory synapses, originating mostly from parvalbumin-positive, fast-spiking interneurons. Such synapses are important for plasticity, as deletion of GABAergic receptors selectively enriched in the synapses between fast-spiking cells and pyramids impairs ODP[55]. At our knowledge, our result that a saturating period of MD similarly affects the ocular preference of excitatory cells and fast-spiking, inhibitory cells is in line with previous reports. Indeed, whereas a short MD period (2 days) has been reported to cause either a lack of[2] or a paradoxical[3] shift of ocular preference in mice (but see[4]), our report of a similar shift of excitatory and inhibitory cells after a saturating MD period is in line with these works. However, this comparison is not straightforward as these works were done in a different species (mouse vs. rat) as well as in a different layer (layers 2/3 vs. 4). However, it is worth mentionining that a study using light-driven c-fos activation reported that parvalbumin-positive cells are selectively refractory to prolonged MD periods compared to excitatory cells[56]. Such a discrepancy might be explained by the use of a more prolonged MD period (more than 150 days vs 10 days), by the fact that the authors of this latter work did not perform a layer-specific analysis, as well as by the different approach.

Thus, our finding that narrow-, fast-spiking units undergo a similar ODP compared to regular-spiking, putative excitatory units suggests that the loss of inhibitory responses observed on excitatory cells is at least partly a presynaptic effect. Because there is a robust thalamocortical innervation of fast spiking cells in layer 4 (e.g.[40], [57], [58]), and because loss of thalamocortical strength is an early event during ODP[36], decreased thalamic input from the deprived eye is a likely cause for the decrease in visual responsiveness in NSUs. However, depression of intralaminar excitatory connections onto NSUs could also play a role.

How general is our finding of a balanced loss of excitation and inhibition after a sensory deprivation? The other main system used to study experience-plasticity *in vivo* is the rodent whisker-to-barrel cortex. While there are no similar studies in the barrel cortex, microiontophoresis data suggested that GABAergic inhibition masks principal whisker responses in the deprived barrels upon rearing animals with only one whisker[59]. However, a slice study using a different deprivation protocol showed that whisker deprivation causes a reduction of intrinsic excitability of layer 4 fast spiking cells[60], accompanied by a loss of synaptic strength of the excitatory connections between layer 4 and layer 2/3[61]. Importantly, such reduction of excitatory drive is accompanied by a similar decrease in the strength of feedforward inhibition[62]. Thus, the excitatory/inhibitory balance in synaptic transmission appeared to be preserved. Similarly, a recent study in vivo study of auditory cortex plasticity in response to activation of cholinergic brain stem nuclei found that excitatory and inhibitory inputs became rebalanced following a temporary imbalance of a few days[63]. Thus, in a variety of primary cortical systems the relative strengths of synaptic excitation and inhibition return to normal levels, following a plasticity-inducing event.

As a final point, it is worth considering why inhibition and excitation decrease in parallel following a saturating period of sensory deprivation. First, a normal excitatory-inhibitory balance is important to guarantee normal sensory processing of inputs coming from the spared sensory pathway (in our case, the left open eye) during cortical map plasticity. Second, it could allow recovery of function in case of re-use of the deprived inputs, because reduced inhibitory transmission is known to favour synaptic potentiation[64] and experience-dependent plasticity in V1[65], [66].
